# Intrafraction 4D‐cone beam CT acquired during volumetric arc radiotherapy delivery: kV parameter optimization and 4D motion accuracy for lung stereotactic body radiotherapy (SBRT) patients

**DOI:** 10.1002/acm2.12755

**Published:** 2019-11-01

**Authors:** Jian Liang, Danielle Lack, Jun Zhou, Qiang Liu, Inga Grills, Di Yan

**Affiliations:** ^1^ Department of Radiation Oncology Beaumont Health System –Royal Oak Royal Oak MI USA; ^2^ Department of Radiation Oncology Beaumont Health System – Troy Troy MI USA; ^3^ Department of Radiation Oncology Emory University School of Medicine Winship Cancer Institute at Emory University Atlanta GA USA

**Keywords:** 4D CBCT, intrafraction, motion validation, SBRT

## Abstract

**Purpose:**

Elekta XVI 5.0 allows for four‐dimensional cone beam computed tomography (4D CBCT) image acquisition during treatment delivery to monitor intrafraction motion. These images can have poorer image quality due to undersampling of kV projections and treatment beam MV scatter effects. We determine if a universal intrafraction preset can be used for stereotactic body radiotherapy (SBRT) lung patients and validate the accuracy of target motion characterized by XVI intrafraction 4D CBCT.

**Methods:**

The most critical parameter within the intrafraction preset is the nominal AcquisitionInterval, which controls kV imaging acquisition frequency. An optimal value was determined by maximizing the kV frame number acquired up to 1000 frames, typical of pretreatment 4D CBCT. CIRS motion phantom intrafraction phase images for 16 SBRT beams were obtained. Mean target position, time‐weighted standard deviation, and amplitude for these images as well as target motion for three SBRT lung patients were compared to respective pretreatment 4D CBCTs. Evaluation of intrafraction 4D CBCT reconstruction revealed inclusion of MV only images acquired to remove MV scatter effects. A workaround to remove these images was developed.

**Results:**

AcquisitionInterval of 0.1°/frame was optimal. The number of kV frames acquired was 567–1116 and showed strong linear correlation with beam monitor unit (MUs). Phantom target motion accuracy was excellent with average differences in target position, standard deviation and amplitude range of ≤0.5 mm. Target tracking for SBRT patients also showed good agreement. Evaluation of phase sorting wave forms showed that inclusion of MV only images significantly impacts intrafraction image reconstruction for patients and use of workaround is necessary.

**Conclusions:**

A universal intrafraction imaging preset can be used safely for SBRT lung patients. The number of kV projections with MV delivery parameters varies; however images with fewer kV projections still provided accurate target position information. Impact of the reconstruction workaround was significant and is mandated for all 4D CBCT intrafraction imaging performed at our institution.

## 1 INTRODUCTION

Image guidance is considered a prerequisite for lung stereotactic body radiotherapy (SBRT) treatment. Among the various image guidance modalities, the linear accelerator gantry‐mounted kilovoltage (kV) cone beam computerized tomography (CBCT) has become one of the most frequently used in‐room imaging technologies. With this system, the CBCT scanner acquires projections over a time period of 70 s to reconstruct a three‐dimensional CBCT volumetric image set (3D CBCT). Unfortunately, the relatively long acquisition time of these 3D CBCT images makes them subject to significant respiratory motion artifacts when imaging the thoracic region.^1^ To address this issue, respiratory‐correlated 4D CBCT has been developed and gradually incorporated into the lung SBRT workflow.^2^, ^3^ In 4D CBCT, the projection images are retrospectively binned according to respiratory phase. Only those x‐ray projections that correspond to the same phase of the patient respiratory trace are reconstructed together, generating multiple phase‐based CBCT datasets which characterize the tumor motion at various phases of the breathing cycle.^1^, ^2^ These 4D CBCT image sets can be directly compared with 4DCT datasets acquired at the time of planning to guide patient setup based on tumor motion.

Elekta has made 4D CBCT commercially available for clinical use through their x‐ray volume imaging (XVI) system (Elekta Oncology Systems Ltd, Crawley, UK), specifically their *Symmetry* VolumeView^TM^ module. With *Symmetry*, the breathing signal necessary for respiratory correlation is extracted from the CBCT projection data itself rather than from an external surrogate^4^, ^5^ or implanted fiducial markers.^6^, ^7^ There are a variety of methods for directly extracting the respiratory trace from the CBCT projections as described in detail by Yan et al.,^8^ however, the method used by the Elekta XVI software is known as the Amsterdam Shroud (AS) method.^1^, ^9^ This method enhances the superior‐inferior motion of the internal anatomy by converting the 2D projection images into an AS image. Once generated, the breathing signal is extracted from the AS image and this signal is used to guide the retrospective binning process necessary for 4D CBCT reconstruction. Accuracy of this method relies on a distinguished moving high‐density anatomical feature such as the patient diaphragm being present in the projection images.^1^, ^8^, ^10^

Parameter settings that direct the XVI software on how to acquire the kV images as well as reconstruct them are specified by what Elekta calls acquisition and reconstruction presets. Optimal parameter settings will vary depending on the anatomical treatment site as well as the type of imaging being acquired (e.g., 3D vs 4D CBCT). Elekta provides its clinical users with a suite of default presets which have been clinically validated and optimized using patient data from their clinical partners.^11^ The ability to use a single preset for multiple patients of a given treatment site and imaging modality is attractive from both a clinical workflow as well as a safety perspective. For 4D CBCT acquisitions, the default preset that is provided by Elekta is called the *Symmetry* preset. This preset has a significantly slower gantry speed than is used for 3D CBCT to ensure that enough projections can be acquired at the different phases of the breathing cycle to generate an accurate AS image, while minimizing artifacts in the multiple phase‐based CBCT datasets. Acquisition times for 4D CBCT images are on the order of 3‐4 vs 1 min for 3D CBCT acquisitions.^12^

The precision requirements of lung SBRT treatment have resulted in institutions acquiring multiple CBCT datasets for each fraction: (a) 4D CBCT before treatment to measure and correct any misalignment of the time‐weighted mean tumor position, (b) 3D CBCT after couch correction to verify the correct couch delta was applied and (c) 4D CBCT after treatment to assess any patient intrafraction motion.^3^ Given the slower acquisition time of 4D CBCT, these additional scans can significantly increase the total treatment time for the patient. Recently, Elekta has introduced a new module to their XVI 5.0.2 software called intrafraction imaging. The XVI kV panel continues to take images at a constant frame rate of 5.5 frame/s during beam on time. Use of intrafraction CBCT allows clinicians to assess patient intrafraction stability without a post‐treatment 4D CBCT and subsequently decreases the total treatment time for the patient. Since the term “intrafraction” imaging has been used in the literature for a variety of imaging techniques in assessing patient intrafraction stability we wanted to define some terms that will be used throughout this work. The term “4D CBCT” will be used to refer to all conventional 4D CBCT acquired without MV delivery. The term “intrafraction 4D CBCT” will be used to refer to 4D CBCT acquisition with the MV beam on.

In order to acquire intrafraction 4D CBCT images, some additional preset parameters need to be defined, one of which is the acquisition interval. This parameter specifies the number of degrees the gantry must move between kV image acquisitions and essentially tells the XVI software when to acquire an imaging frame which will be used for reconstruction. This parameter combined with the gantry speed control the number of imaging frames that the XVI software can acquire per second up to the limit set by the kV detector panel.^13^ A second preset parameter that will be affected during intrafraction imaging is the gantry speed setting. Previously, it was mentioned that for the *Symmetry* preset, a significantly slower gantry speed was chosen to ensure that enough imaging frames were acquired at each phase of the breathing cycle to generate an accurate 4D CBCT image set. However, with intrafraction imaging, this setting must be disabled to allow the MV delivery beam settings to control the gantry speed. This poses a challenge for intrafraction 4D CBCT imaging, where the gantry speed for certain treatment deliveries may result in significant differences in the angular spacing between projections as well as an inadequate number of projections for each phase of the breathing cycle. Large and irregular angular spacing between projections has been noted to be a problem for conventional 4D CBCT acquisitions that have a uniform gantry speed. These irregularities are attributed to the sinusoidal nature of patients breathing curves and the variations in time that a patient spends in each of the 10 phases of their breathing cycle combined with the amount of gantry movement that occurs between projections at a given phase of the cycle. This results in projection clustering for some phases of the breathing cycle and missed projections for other phases.^14^, ^15^ To get around this challenge, individual intrafraction presets may need to be set for each SBRT lung patient in order to ensure an that appropriate intrafraction 4D CBCT image can be generated. This poses some clinical workflow and safety challenges, resulting in a very large database of image acquisition presets for the therapists to select from and the potential risk of choosing an incorrect preset. Another challenge to intrafraction imaging is the effects of MV scatter photons on the image quality, as the MV beam is on during kV imaging^16^. To address this, there have been a number of potential solutions which have been proposed.^17^, ^18^, ^19^, ^20^ Specifically, for XVI 5.0.2, Elekta has implemented a correction method where images are acquired on the kV panel during treatment delivery with the kV x‐ray tube off. The MV scattered radiation noise and artifacts acquired during these MV only images are then subtracted from the kV‐MV imaging frames at nearby gantry angles. By subtracting out the noise and artifacts caused by the treatment beam, Elekta states that the acquired intrafraction images are almost the same kV image acquired without the MV treatment beam on.^13^, ^21^

In this work, we use a commercial phantom to systematically validate the accuracy of target motion determined by the Elekta XVI intrafraction 4D CBCT imaging module for SBRT lung patients. To date, most of the previous reports on intrafraction CBCT have been focused on the feasibility and development of this technology and evaluation of diagnostic image quality parameters (e.g. contrast‐to‐noise ratio).^17^, ^18^, ^19^, ^20^, ^22^, ^23^ While there have been a few reports that have discussed the clinical implementation of this technology,^24^, ^25^, ^26^ none of these studies have evaluated the accuracy of target tracking to the detail that is presented in this work. We also investigate whether a standard universal intrafraction imaging preset could be used for all SBRT lung patients by evaluating preset parameters for a wide range of arc lengths and monitor unit (MU) deliveries. Lastly, we evaluate the target motion determined from intrafraction 4D CBCT phase images for a small cohort of SBRT lung patients treated in our clinic.

## 2 MATERIALS AND METHODS

### 2.A Patient data

Treatment plans from 16 SBRT lung patients previously treated in 2017 at our institution were selected for this IRB‐approved retrospective study. All patients were treated on an Elekta Synergy Beam Modulator machine (Elekta, Stockholm, Sweden) with 2 volumetric modulated arc therapy (VMAT) beams with arc lengths ranging from 150‐200 degrees and monitor units (MUs) ranging from 842 to 2084 per beam. Since our clinical workflow would involve acquiring intrafraction 4D CBCT images during delivery of the second treatment beam, the second treatment beams from each patient plans were used to evaluate imaging preset parameters described in II.B. and to validate the intrafraction imaging module described in II.C. for lung SBRT treatments.

### 2.B Development of intrafraction imaging preset for XVI

In order to use the XVI intrafraction imaging module, an acquisition preset needed to be created. Since the goal of this study was to validate intrafraction imaging for SBRT lung patients, the 4D CBCT *Symmetry* preset was copied and modified as per vendor recommendations. Modifications included removal of the gantry speed setting from the preset, adjusting the gantry start and stop angles for image acquisition, and the addition of three new parameters (as described below) that are specific to intrafraction imaging.^11^, ^13^, ^21^ The first parameter IntrafractionImaging had to be set as Yes to enable the intrafraction imaging function. The second parameter StaticMaxDuration allows the user to specify the amount of time at the start of an imaging acquisition where the gantry remains stationary and acquires 2D planar kV images. It was set to 4 s which is the lowest value allowed by the vendor. The last parameter AcquisitionInterval, is the minumum gantry movement between two KV projection image acquisition. The maximum value for this parameter is 9.0, and the minimum value is 0.1. During intrafraction imaging process, the imaging panel took image signals at a constant rate of 5.5 frames/s. When the gantry does not move fast enough at certain angles, a kV source off (MV only) projection image would be taken instead.^13^

Optimal setting of the AcquisitionInterval parameter is important to ensure that enough frames are acquired to generate clinically acceptable intrafraction images while limiting the kV dose to the patient. Four choices of setting AcquisitionInterval to 0.1, 0.2, leaving the value blank (e.g. AcquisitionInterval=), and removing the parameter line from the preset text file were initially tested for two patient beams from the SBRT test suite representing the smallest (842) and largest (2084) MU treatment deliveries. The effect of this setting on the number of imaging frames generated was evaluated with a goal to achieve as many imaging frames as possible with an upper limit close to 1000 imaging frames which is the amount of frames routinely acquired with our 4D CBCT *Symmetry* preset. This would ensure that the imaging dose for intrafraction 4D CBCT images would be similar or less than what we have been delivering clinically for our post‐treatment 4D CBCT image acquisitions.

For the beam with the smallest MU, all settings tested for AcquisitionInterval = 0.2, 0.1, Blank, and removing the entire parameter line resulted in a total number of kV projections of 508, 567, 685 and 668 frames respectively. While all of these projection values were less than 1000, it was noted that when this setting was removed or left blank, more kV projections were acquired at the kV starting acquisition angle prior to the start of treatment delivery (21 projections acquired) vs. when an AcquisitionInterval of 0.2 or 0.1 was used (14 kV projections acquired). Additionally, when the AcquisitionInterval was left blank fewer kV projections were acquired between the MV stop angle and kV stop acquisition angle. This was not seen for any of the other parameter settings. For the largest MU beam (2084 MU), setting values of 0.1 and leaving the value blank were tested further. While the total number of kV projections acquired for a setting of 0.1 was 1116, close to the target value of 1000, the total number for a blank setting was significantly higher at a value of 1552 frames. Given that use of a blank AcquisitionInterval setting: 1) increased the number of static kV projections acquired at the gantry start angle, 2) resulted in fewer projections acquired at the end of MV delivery, and 3) produced too many projections for large MU beams, a value of 0.1°/frame was deemed optimal for use in each of the intra‐fraction imaging presets that would be used in this study.

As per our past clinical gantry setting for most lung SBRT patient plans, in total four intrafraction presets as shown in Table 1 were created in our institution for SBRT to allow imaging of right and left sided lung tumors and to scan clockwise and counter‐clockwise. Those preset names must be added to machine characterization within Mosaiq system. This allows for each of the presets to be automatically selected for use within the Mosaiq/XVI Synergistiq system when a beam was selected.

**Table 1:** Gantry start and stop angles in XVI intrafraction imaging presets for lung stereotactic body radiotherapy.

Treatment site	Direction	XVI preset name	Start angle (°)	Stop angle (°)
Right side	CW	ta_Intrafraction_RtLung‐CW	−179	21
	CCW	tb_Intrafraction_RtLung‐CC	22	−178
Left side	CW	tc_Intrafraction_LtLung‐CW	−22	178
	CCW	td_Intrafraction_LtLung‐CC	179	−21

For all presets, the start gantry angle also is the start acquisition angle for kV imaging.

### 2.C CIRS phantom and validation of Elekta intrafraction imaging module

**Figure 1:**
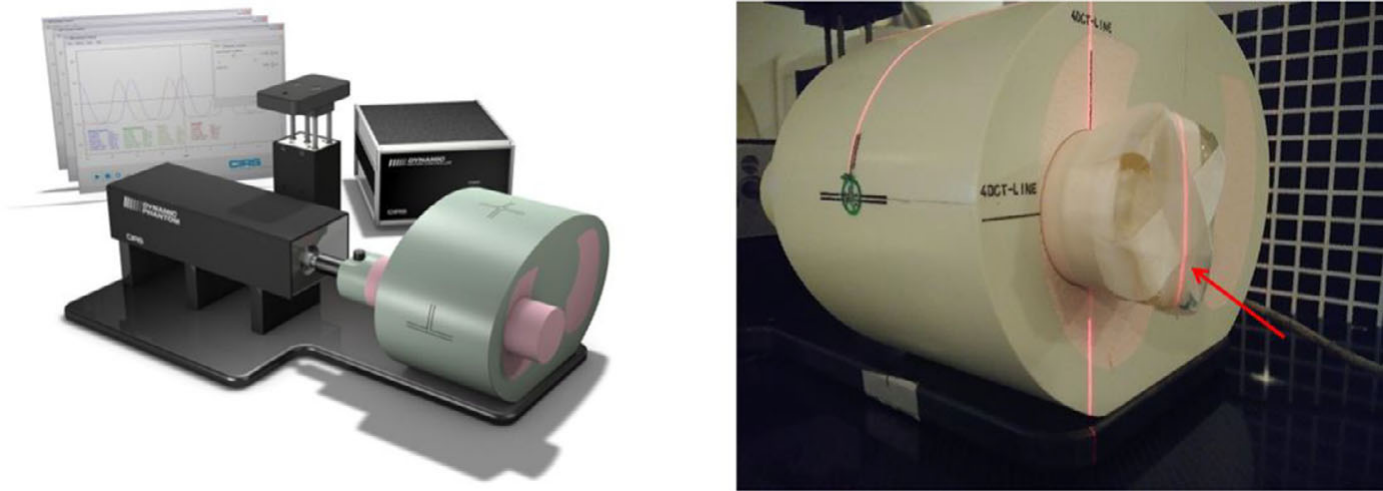
CIRS dynamic thorax phantom. Left: thorax phantom with lung equivalent cylindrical rod, motion actuator box and surrogate platform is shown. Right: phantom with 1 cm bolus modification is shown and highlighted by red arrow.

Accuracy of target motion as determined from intrafraction 4D CBCT imaging was assessed using the CIRS Dynamic Phantom (CIRS, Norfolk, VA) shown in Fig. 1. A 1 cm thick bolus was attached to the free end of the lung equivalent cylindrical rod within the phantom (as indicated by the red arrow in Fig. 1) to simulate the high density region of a patient's diaphragm. This will help the XVI software to improve the phase sorting accuracy.

**Figure 2:**
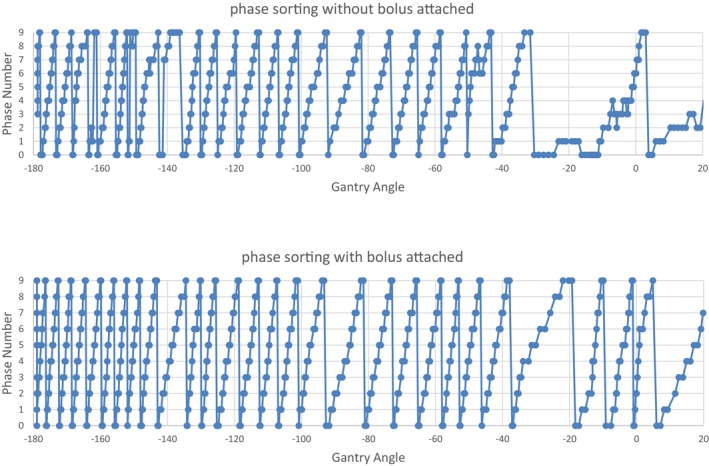
Difference in phase sorting of intrafraction four‐dimensional cone beam computed tomography imaging using the CIRS phantom with and without a high‐density bolus attached for an 842 MU beam. Image projection angle = Gantry angle + 90.

The impact of this bolus addition on the intrafraction 4D CBCT phase sorting process is shown in Fig. 2. Without the bolus, discrepancies of phase sorting can be seen in the projections when the gantry angles were around −160° and near −40° to 5°.

For this study, two different sized spherical targets with diameters of 10 mm (S10) and 20 mm (S20) were used and are representative of the typical tumor sizes treated for SBRT lung patients.^27^, ^28^ The target material was soft‐tissue equivalent with linear attenuation within 1% of water.Target motion was programmed to follow a sinusoidal pattern as described in Eq. (1), which represents a normal patient breathing trace. In Eq. (1), Z(t) is the position of the target in the superior‐inferior direction at time t, A represents the motion amplitude in mm and t represents the time in seconds.^29^ Motion amplitudes of 10 mm (M10) and 20 mm (M20) were evaluated and a breathing period of 4 s was selected.

(1) $$Z\left(t\right)=A\cos4\frac{\pi }{4}t$$

To get reference data sets for registration of 4D CBCT images, the phantom was scanned on a Philips Big Bore CT scanner using our clinical 10‐phase 4DCT protocol in conjunction with the Philips bellows system (Philips Healthcare, Bothell, WA) for each of the four phantom setups (S10M10 = tumor size 10 mm with motion amplitude of 10 mm, S10M20 = tumor size 10 mm with motion amplitude 20 mm, S20M10 = tumor size 20 mm with motion amplitude 10 mm, S20M20 = tumor size 20 mm with motion amplitude 20 mm). The mean‐position phase image was chosen as the reference image. The gross tumor volume (GTV) on each of the 10 phase images was delineated, and the internal tumor volume (ITV) was generated from the 10 GTVs. This generated ITV was copied to the mean‐position phase image set. All 16 patient treated beams were copied under the reference image. The isocenter was set to the center of ITV. Those beams, along with GTV and image on the mean‐position phase as well as ITV formed the reference dataset in XVI database.

An initial 4D CBCT was acquired in XVI using the *Symmetry* preset. Phantom setup position was adjusted using a dual registration method where alignment was focused on the target ITV region. After couch correction, a second verification 4D CBCT was acquired and registered in XVI. The target position exhibited on this verification 4D CBCT was used as the reference for comparison with intrafraction imaging registration data eliminating any discrepancies in target positioning that may have been caused by phantom setup error.

**Figure 3:**
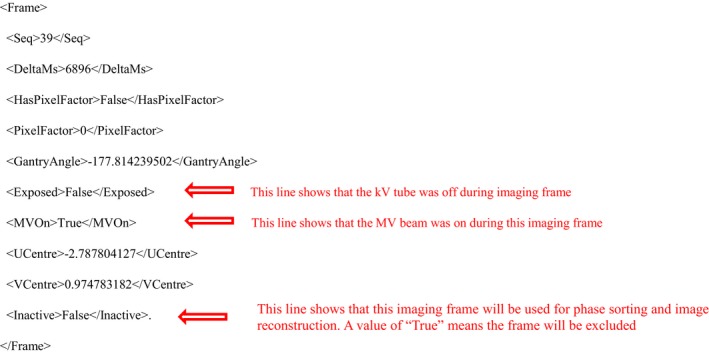
Snippet of XVI internal data file that specifies what imaging frames were acquired and if they will be used for image reconstruction and phase sorting.

Each beam was delivered four times to capture imaging for the four combinations of phantom setups being tested. For each CBCT session, an internal XML data file, _frames.xml was generated before xvi starting for phase sorting process. This file contains information related to each imaging frame captured by the kV imaging panel during acquisition. A snippet of the text from one of these data files is shown in Fig. 3. Here data are presented for imaging frame 39 and it can be seen that the imaging frame captured is an MV only image (MvOn = True, Exposed = False) which is set to be included in image reconstruction and phase sorting (Inactive = False). It seems that those MV only images were not excluded in phase sorting because of the tag Inactive = False were defined in the internal file. Further study verified our observation and an in‐house program was developed to modify it such that all MV only images are excluded (by setting Inactive = True) from the phase sorting and image reconstruction process.

To implement this workaround to the intrafraction 4D CBCT reconstruction process, reconstruction was cancelled following intrafraction image acquisition and the in‐house software was used to review and modify the XVI internal imaging file _Frames.XML accordingly. Once this modification was completed, reconstruction was performed using the same reconstruction preset as defined in 4D *Symmetry*. A dual (bone followed by soft tissue) registration method was followed. Manual registration was performed when necessary. Target position on each of the 10 CBCT phases was recorded to compute the mean target position and motion amplitude/range for comparison with the verification 4D CBCT registration results. Additionally, the shape of the target motion curve constructed from 10 intrafraction phase images was compared to that corresponding curve from the verification 4D CBCT image sets. Accuracy of target motion shape derived from the intrafraction was assessed using a time‐weighted standard deviation calculation. Assuming that the tumor position at the i‐th phase image is Z*i* and the total acquisition time for all the kV images in the i‐th phase is T*i*. The time‐weighted mean position, Z*ave* can be calculated as,

(2) $$Zave=\left(\sum Zi*Ti\right)/\sum Ti$$

and the time‐weighted standard deviation can be calculated as,

(3) $$Std=\sqrt{\left(\sum Zi*Zi*Ti\right)/\sum Ti-Zave*Zave}$$

### 2.D Patient data validation

The intrafraction 4D CBCT imaging module was tested further for three of the 16 SBRT lung patients in our test suite. Patients were selected based on two criteria (a) they had a tumor excursion of 10 mm or more and (b) their treatment beam monitor units were representative of the smallest, middle and largest values evaluated in our test suite. For patient images, the reconstruction workaround described in II.C was also used. Unlike the phantom which provides a reproducible sinusoidal breathing pattern over the entire course of image acquisition, patient breathing traces can vary significantly and there is no gold standard for the mean detected target position on any 4D CBCT. Therefore, to assess intrafraction imaging for these patients, only the target motion shape and amplitude were compared between positions exhibited on the pretreatment 4DCBCT and intrafraction 4D CBCT.

### 2.E Impact of intrafraction 4D CBCT reconstruction workaround

To determine if the inclusion of these purely MV imaging frames could cause phase sorting and reconstruction problems, we compared the intrafraction 4D CBCT imaging results and phase sorting waveforms with and without the XVI _frames.xml file being modified for both phantom and patient images. Since there is no reference respiratory trace to compare to for patient images, a gold standard was created by manually delineating the diaphragm on every kV projection of the intrafraction 4D CBCT dataset within XVI and making the assumption that the target volume and diaphragm have the same phase information for each projection. Diaphragm and XVI phase sorting information waveforms had to be scaled down to allow them to be compared on a single plot. Scaling was performed as shown in Eq. (4) for the XVI phase sorting waveform,

(4) $$PhaseID=\left\{\begin{array}{c} p,\;\mathrm{if}\;p=0,1,2,3,4\\ 10-p,ifp=5,6,7,8,9 \end{array}\right.$$

where *p* represents the sorting phase index number between 0–9 and as shown in Eq. (5) for the diaphragm position waveform,

(5) $$DiaphragmPosition=4.0\times \frac{\left(P-\text{Pmin}\right)}{\left(\text{Pmax}-\text{Pmin}\right)}$$

where P is the measured top position of the diaphragm from a given imaging frame and Pmax and Pmin represent the maximum and minimum diaphragm positions observed for that intrafraction image. Additionally, to evaluate the impact of XVI phase sorting on diaphragm tracking for patient images, reconstructed intrafraction 4D CBCT phase image quality was compared (with and without modification of the XVI internal file).

## 3 RESULTS

### 3.A Evaluation of acquisition interval preset setting

**Figure 4:**
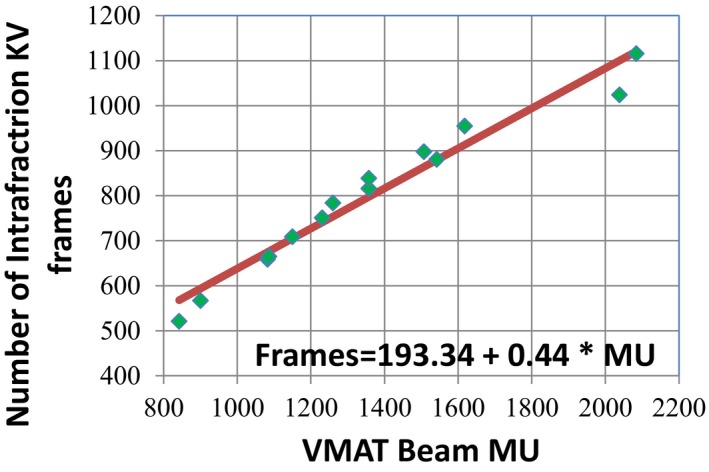
Correlation of total number of kV frames acquired with intrafraction imaging as a function of volume modulated arc therapy treatment beam MUs.

Despite a preset setting of 0.1°/frame, the actual acquisition interval achieved for intrafraction imaging varies throughout image acquisition and is a function of the gantry speed and the max kV panel frame rate of 5.5 frames/s. For the 16 SBRT lung treatment beams tested, the average actual acquisition interval obtained ranged from 0.18°/frame to 0.35°/frame for the largest and smallest MU beams respectively. For all of the beams tested, the total number of kV projections acquired ranged in value from 567 to 1116 with an average value of 781 ± 162 projections. Figure 4 demonstrates that the total kV frame number showed a strong linear correlation with the treatment beam MU with an R^2^ value of 0.96.

### 3.B Motion validation using CIRS phantom

The phantom target motion accuracy determined with the Elekta intrafraction imaging module for each of the 16 SBRT treatment beams is summarized in Table 2. Differences in calculated target mean position, position standard deviation (SD), and amplitude for each of the intrafraction 4D CBCT images as compared to the reference 4D CBCT images are presented for the four phantom setups tested. In total, the target mean position uncertainty for intrafraction imaging for all phantom setups and all treatment beams was sub‐mm with an average difference of −0.1 ± 0.3 mm and range in values between −0.7 and 0.8 mm. Specifically, for each of the four phantom setups the average uncertainty in the mean target position was −0.2 ± 0.2 mm, 0.3 ± 0.3 mm, −0.2 ± 0.1 mm, and −0.1 ± 0.1 mm for S10M10, S10M20, S20M10, and S20M20 respectively. The total measured amplitude uncertainty was also sub‐mm with an average difference of −0.1 ± 0.5 mm, however the range was greater than 1 mm with values between −1.1 and 1.5 mm. Average differences for each of the four setups were −0.4 ± 0.4 mm for S10M10, 0.0 ± 0.5 mm for S10M20, −0.2 ± 0.2 mm for S20M10, and 0.3 ± 0.6 mm for S20M20.

**Table 2:** Measured uncertainty in target phase position determined from intrafraction imaging in CIRS phantom(mean, time‐weighted standard deviation (STD), amplitude).

Beam information			Mean position difference (mm)				STD difference (mm)				Amplitude difference (mm)			
Beam #	MU	MV‐span	S10M10	S10M20	S20M10	S20M20	S10M10	S10M20	S20M10	S20M20	S10M10	S10M20	S20M10	S20M20
1	842	180	−0.1	0.6	0.0	−0.1	−0.1	0.0	0.0	0.1	−0.6	0.8	−0.2	0.6
2	900	164	−0.2	0.3	−0.2	−0.1	0.1	−0.2	0.0	−0.2	0.2	−0.1	−0.1	−0.8
3	904	150	−0.2	0.4	−0.2	−0.4	0.1	0.1	0.0	0.1	−0.1	−0.4	0.1	0.1
4	1082	180	−0.4	0.3	−0.3	−0.1	−0.3	0.3	0.0	0.1	−0.3	−0.1	0.1	0.8
5	1086	179	−0.6	0.6	−0.3	−0.2	−0.1	0.1	0.0	−0.1	0.2	−0.2	0.1	−0.8
6	1137	180	−0.2	0.8	−0.2	−0.1	−0.1	0.5	0.0	0.1	−0.5	−0.8	−0.3	0.7
7	1150	180	−0.1	0.7	−0.2	−0.1	−0.1	0.0	0.0	0.1	−0.3	0.2	−0.3	0.6
8	1218	170	0.1	0.1	−0.1	−0.2	−0.2	0.5	0.0	0.3	−1.1	−0.6	−0.2	1.5
9	1231	169	−0.2	−0.3	−0.1	−0.1	−0.5	0.0	0.0	−0.1	−0.7	0.5	0.0	0.2
10	1260	150	0.0	0.1	−0.2	−0.3	0.1	−0.2	0.0	0.2	0.3	0.8	−0.4	0.4
11	1270	180	−0.7	−0.3	−0.2	−0.3	−0.4	0.2	0.0	0.0	−0.5	0.0	−0.1	0.3
12	1357	200	−0.1	0.5	−0.1	−0.1	−0.3	0.2	0.1	0.1	−1.0	0.4	−0.2	0.6
13	1357	180	−0.1	0.1	−0.2	0.0	−0.2	0.3	0.0	−0.1	−1.0	−0.4	0.1	−0.6
14	1507	180	−0.2	0.2	−0.3	−0.1	−0.1	0.0	−0.1	0.1	−0.8	0.0	−0.6	0.6
15	1618	180	0.0	−0.1	−0.2	0.0	0.0	−0.1	0.1	0.0	−0.5	−0.7	−0.3	0.5
16	2084	200	0.2	0.0	0.0	0.0	0.0	0.1	0.0	0.0	−0.2	0.2	−0.3	0.3
Minimum	842	150	−0.7	−0.3	−0.3	−0.4	−0.5	−0.2	−0.1	−0.2	−1.1	−0.8	−0.6	−0.8
Maximum	2084	200	0.2	0.8	0.0	0.0	0.1	0.5	0.1	0.3	0.3	0.8	0.1	1.5
Average	–	–	−0.2	0.3	−0.2	−0.1	−0.1	0.1	0.0	0.1	−0.4	0.0	−0.2	0.3
STDev	–	–	0.2	0.3	0.1	0.1	0.2	0.2	0.0	0.1	0.4	0.5	0.2	0.6
Reference CBCT 1			–	–	–	–	3.6	7.0	3.5	7.1	10.1	19.8	9.8	19.7
Reference CBCT2			–	–	–	–	3.5	6.9	3.5	7.1	9.6	19.4	9.5	19.8
Nominal value			–	–	–	–	3.7	7.4	3.7	7.4	10.0	20.0	10.0	20.0

S10M10: nominal target size = 10 mm diameter of sphere, nominal motion amplitude = 10 mm.

S10M20: nominal target size = 10 mm diameter of sphere, nominal motion amplitude = 20 mm.

S20M10: nominal target size = 20 mm diameter of sphere, nominal motion amplitude = 10 mm.

S20M20: nominal target size = 20 mm diameter of sphere, nominal motion amplitude = 20 mm.

**Figure 5:**
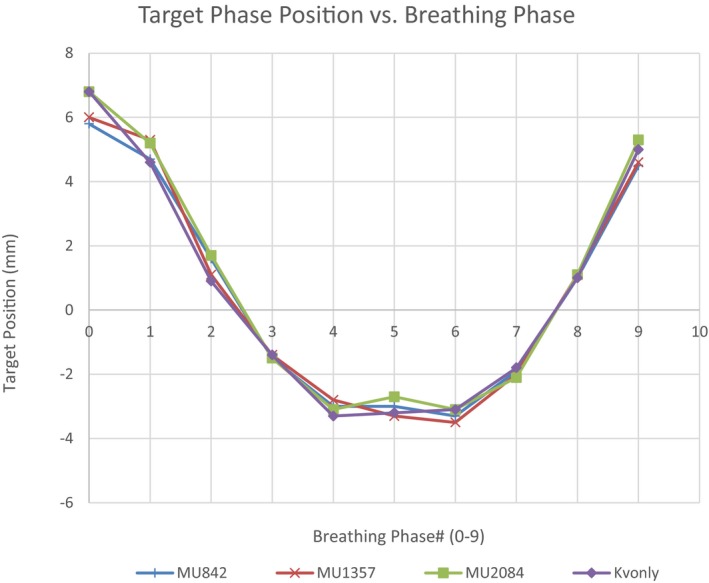
Target tracking for each of the 10 phases for the S10M10 phantom setup for three different treatment beams as well as the reference four‐dimensional cone beam computed tomography (kV only).

Figure 5 presents the accuracy of intrafraction 4D CBCT imaging to correctly determine the target motion shape (target position vs. breathing phase) for three treatment beams representing the smallest, middle and largest MUs compared to the reference 4D CBCT. Results are shown for the phantom setup with the smallest target and motion amplitude (S10M10). Target motion curves were reviewed for all of the beams tested as well as all four phantom setups and found to be similar in shape to the reference 4D CBCT. A time‐weighted standard deviation calculation was used to quantify the differences in target motion shape compared to the reference 4D CBCT for all beams and each of the phantom setups. Results are presented in Table 2 where it can be seen that the total standard deviation uncertainty for intrafraction imaging was also sub‐mm with an average difference of 0.0 ± 0.2 mm and a range in values between −0.5 and 0.5 mm. Average differences for each of the phantom setups S10M10, S10M20, S20M10, S20M20 were −0.1 ± 0.2 mm, 0.1 ± 0.2 mm, 0.0 ± 0.0 mm, and 0.1 ± 0.1 mm respectively.

For reference, Table 2 also shows the calculated standard deviation and amplitude results from the reference 4D CBCTs from each of the four phantom setups as well as the nominal values calculated from Eq. (1). It should be noted that intrafraction 4D CBCT phantom measurements were taken over two separate days resulting in the two reference 4D CBCT data sets with values shown in Table 2. Comparatively, the reference 4D CBCT data sets show good agreement with the nominal calculated values with a max difference of 0.6 mm seen for the nominal amplitude with the S10M20 phantom setup.

**Figure 6:**
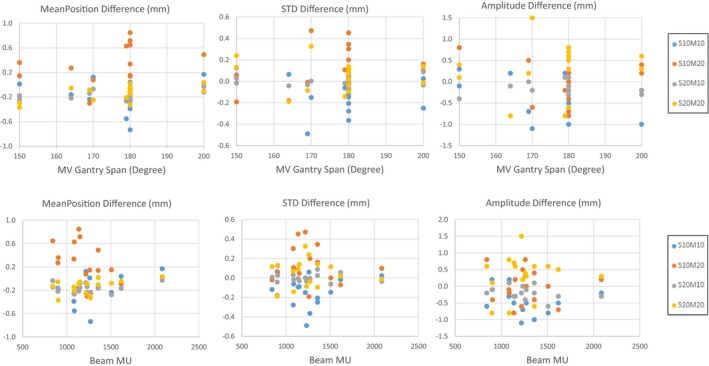
Relationship between target motion uncertainty in mean position, standard deviation and amplitude as a function of MV treatment beam gantry span, and MV treatment beam MUs for each of the four phantom setups (S10M10, S10M20, S20M10, S20M20). The Y‐axis title = chart title.

Plots of the measured uncertainty in phantom target tracking (mean position, standard deviation, and amplitude) as a function of treatment beam gantry span [Fig. 6(a)] and treatment beam MU [Fig. 6(b)] for all the treatment beams and phantom setups tested are presented. Measured uncertainty for all parameters was less correlated with treatment beam gantry span than with treatment beam MUs where beams with >1500 MUs showed smaller target tracking uncertainty. The maximum absolute uncertainty for the mean target position, standard deviation and amplitude was 0.3, 0.1 and 0.8 mm for beams with> 1500 MU respectively. For beams with <1500 MU the max absolute uncertainties for each of these parameters were 0.8, 0.5 and 1.5 mm respectively.

**Figure 7:**
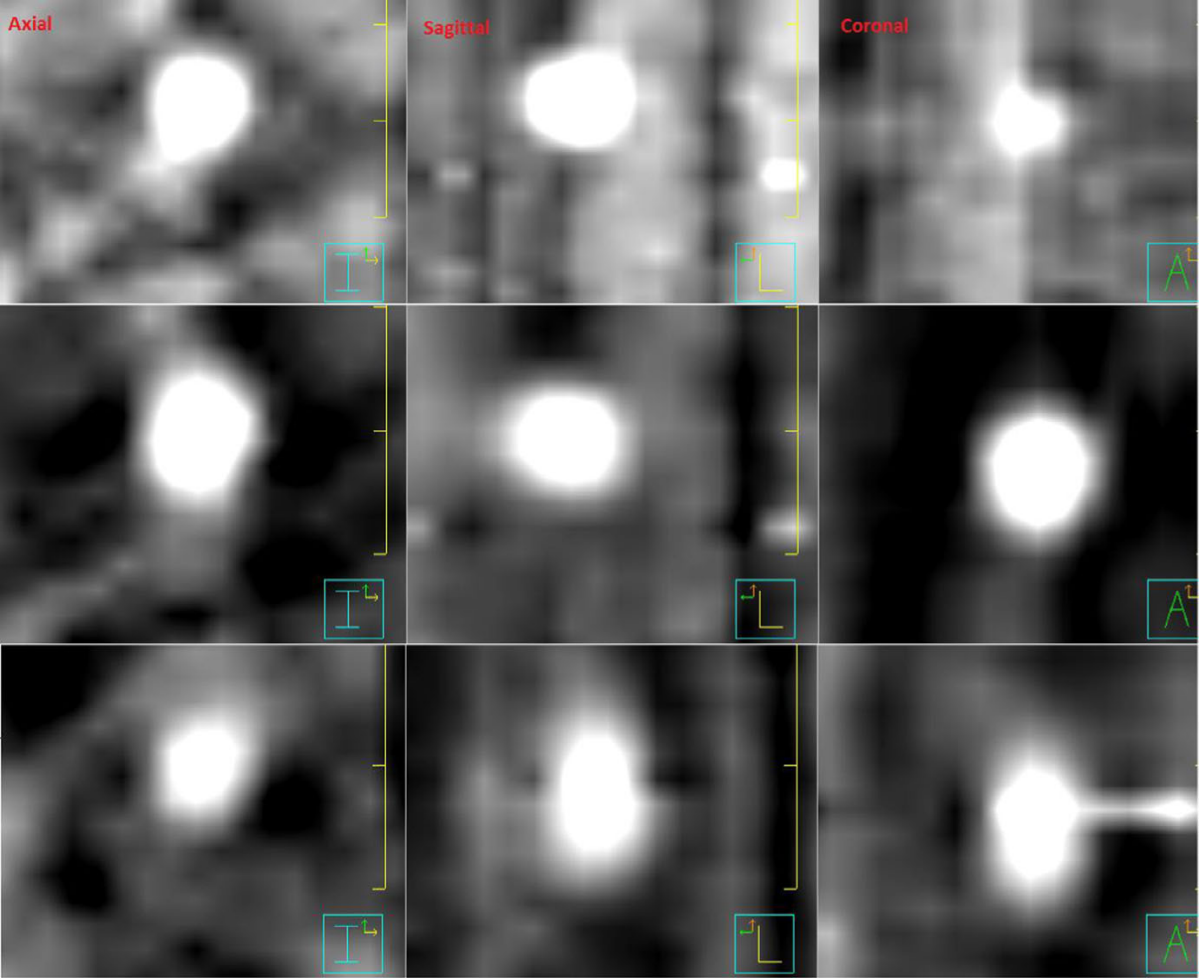
Intrafraction four‐dimensional cone beam computed tomography reconstructed S10M20 phantom images for three breathing phases (Top‐row: phase 0, Mid‐row: phase 9, Bottom‐row: phase 3). Phantom target is a sphere and should appear as a circle on reconstructed phase images. Deformation of target shape as a result of unevenly spaced and reduced amount of kV projections can be seen. The Window and Level settings used in the Pinnacle Planning system were: 259 and 238.

The number of kV projections in intrafraction images varies as described in III.A. Compared to pretreatment or post‐treatment 4D CBCT images which have about 1000 projections with uniform gantry spacing, the un‐uniform gantry spacing and overall lower number of projections can affect the image quality of the 4D CBCT. This is shown in Fig. 7 which presents S10M20 phantom phase images from an intrafraction 4D CBCT with 567 kV projections. Deformation of the spherical tumor shape which should appear as a circle on any given phase slice in the axial, sagittal and coronal directions can be seen.

### 3.C Patient Data Validation

**Figure 8:**
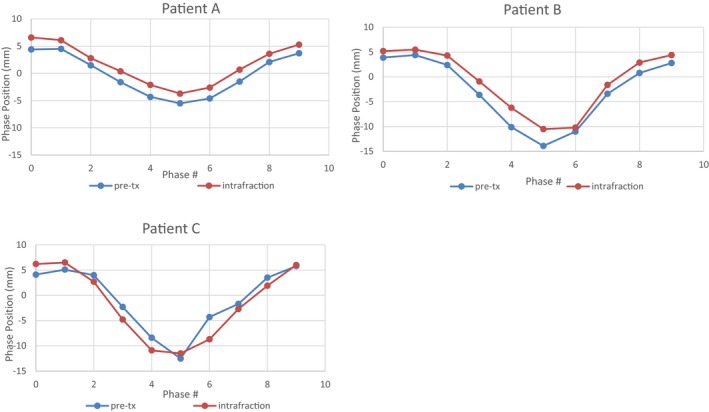
Target tracking for each of the 10 phases for each of the three stereotactic body radiotherapy patients for the pretreatment four‐dimensional cone beam computed tomography (4D CBCT) and intrafraction 4D CBCT. 0 = exhale phase, 5 = inhale phase. Phase position zero line corresponds to the treatment machine isocenter position.

Table 3 presents the target motion results for a single fraction for each of the three SBRT patients evaluated in this study. The difference in tumor mean position between the pretreatment 4D CBCT and the intrafraction 4D CBCT represents target motion during treatment delivery. Target tracking for each of the three SBRT patients is presented in Fig. 8 and demonstrates the similarity in the target motion shape with respect to amplitude and standard deviation observed for the pretreatment and the intrafraction 4D CBCTs. From Table 3, the largest difference in amplitude between the pretreatment and the intrafraction 4D CBCTs was 2.3 mm for patient B, and standard deviation differences were <1 mm for all three patients.

**Table 3:** Patient intrafraction target motion results for each of the three stereotactic body radiotherapy (SBRT) patients evaluated in this study.

Patient	Beam MU/MV gantry span (°)	Pretreatment 4D CBCT amplitude/Std (mm)	Intrafraction 4D CBCT amplitude/Std (mm)	Difference amplitude/Std (mm)	Mean position shift between images (mm)
A	1618/180	10.0/3.9	10.3/3.7	0.3/−0.2	1.7
B	1218/170	18.3/6.6	16.0/6.3	−2.3/−0.3	1.7
C	900/164	18.2/6.1	18.1/7.0	−0.1/0.9	2.1

### 3.D Impact of intrafraction 4D CBCT reconstruction workaround

**Figure 9:**
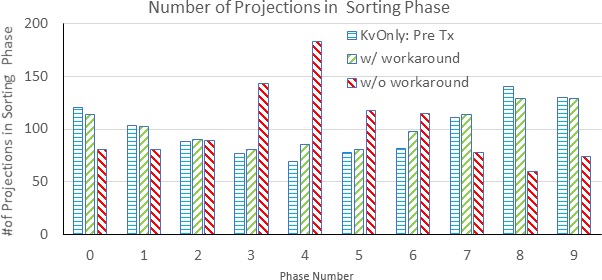
Distribution of two‐dimensional projection images in each sorting phase.

Phantom and patient intrafraction 4D CBCT imaging results with and without the reconstruction workaround were compared. For the phantom imaging, phase sorting errors in the un‐modified XVI files occurred for a very limited range of kV projection angles, hence the reconstructed 4D CBCT image quality wasfound to be similar for images reconstructed with and without the workaround. Despite what was seen for the phantom images, large discrepancies in phase sorting were observed for the patient images. For example, Fig. 9 presents the number of projections per sorting phase for (a) a kV only 4D CBCT and an intrafraction 4D CBCT (b) without the workaround and (c) with the workaround. Without the workaround, incorrect phase sorting results in an uneven distribution of frames between phases with 98 frames being incorrectly sorted into Phase 4. This uneven distribution of imaging frames between phases is resolved with the workaround in place.

**Figure 10:**
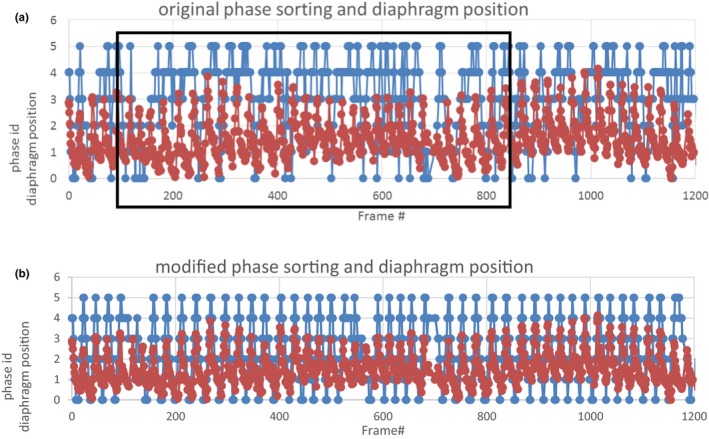
(a) Original XVI phase sorting (blue curve) and manually detected diaphragm position (red curve) wave forms for a single stereotactic body radiotherapy lung patient. The black box highlights frames with phase sorting errors. (b) Modified phase sorting (blue curve) and manually detected diaphragm position (red curve) wave forms after modification of internal XVI file _frames.xml.

Figure 10(a) and 10(b) presents the diaphragm position and XVI‐sorted phase information for a single SBRT patient with and without modification of the internal XVI file. Compared with the manually detected diaphragm position waveform, Fig. 10(a) shows many phase sorting errors for the original XVI waveform especially between frames 180‐800. With the modification to the internal XVI file, the results are much improved as shown in Fig. 10(b).

**Figure 11:**
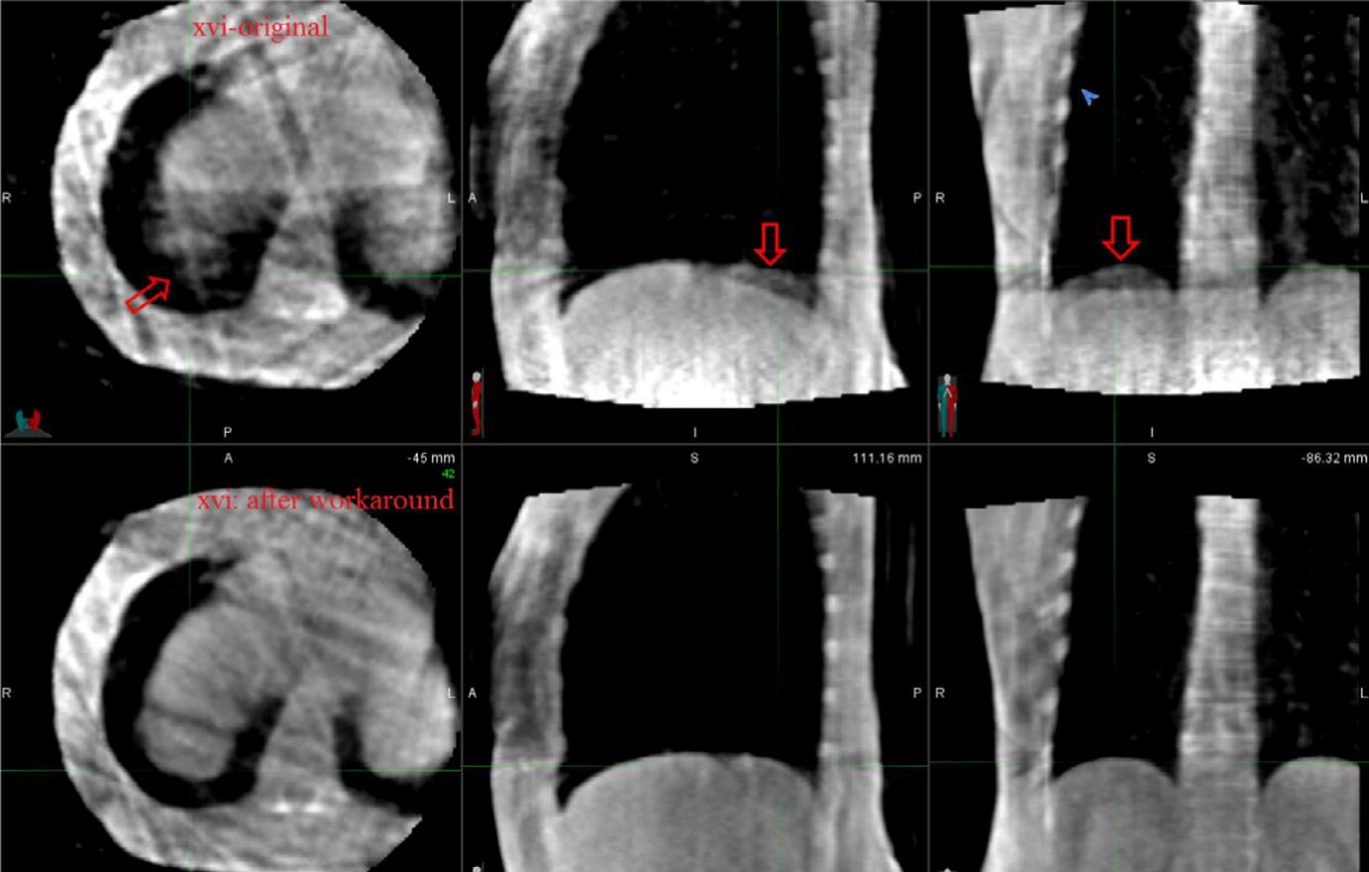
Comparison of intrafraction four‐dimensional cone beam computed tomography images for a stereotactic body radiotherapy lung patient with and without XVI file modification workaround. Same Window and Level was applied for both image displays. Without workaround there is significant blurring of the diaphragm position highlighted by the red arrows making tracking of the diaphragm position difficult.

Figure 11 shows the image quality of the 4D CBCT phase images for the above patient case compared with and without workaround.

## 4 DISCUSSION

Elekta has provided its clinical users with a suite of validated clinical presets to use when imaging their patients with the XVI system for 3D Volume View and conventional 4D *Symmetry* image acquisitions. The ability to use a universal preset for all patients of a given treatment site greatly simplifies clinical workflow and reduces potential errors that could arise from having to manually create, associate and select a different preset for each patient. This also ensures that the images generated with that preset will be optimal for guiding patient setup for that specific treatment site, reducing the need to take subsequent CBCT images.

When implementing intrafraction imaging, the question arises as to whether a universal preset can be used since the gantry speed for intrafraction 4D CBCT acquisition depends on the gantry speed required for MV delivery. Since MV delivery gantry speeds vary depending on the complexity of the SBRT lung plan, it is quite possible that use of a standard intrafraction imaging preset could result in suboptimal 4D CBCT images for some patients.

In this study, we evaluated different settings for the AcquisitionInterval parameter. Elekta recommends not setting this parameter for 4D CBCT acquisitions to prevent the clinical user from using a value that is too large and generating images with not enough imaging frames. However, we found that leaving this value blank was also suboptimal for a few reasons (a) a higher number of static kV images were taken at the start of image acquisition resulting in additional imaging dose to the patient without generating additional useful image information, (b) some imaging frames were not captured, specifically for the gantry angles that directly followed the end of MV delivery (over a 2°–2.5° range) and (c) for large MU treatment beams (~2000 MU), the number of imaging frames acquired was significantly greater than our target number (~1000) acquired for conventional 4D CBCT image acquisitions, meaning a higher imaging dose would be delivered to the patient.

Instead of leaving this value blank, we found that a setting of 0.1°/frame was optimal for large MU treatment beams in terms of the number of kV projections generated. Based on the measured uncertainty results for determining target phase position shown in Table 2, this value was also adequate for small MU beams (842 MUs), where all of the measured uncertainty (mean position, standard deviation, and amplitude) results were 0.8 mm or less. This suggests that a universal intrafraction imaging preset for 4D CBCT acquisition can be used at our clinic for patients receiving SBRT lung treatment. Figure 4 presents the correlation between the number of intrafraction kV imaging frames and the treatment beam MUs of which there was a strong correlation. This correlation, although based on a small sample of treatment cases can be used to quality assurance future SBRT lung treatment beams in our clinic by providing us with an estimate of the number of imaging frames that will be generated. Clinically, we have implemented this model into a daily monitor process which reports the number of imaging frames for any new patients who will receive intrafraction imaging and checks that the number is within 550–1100 frames. This check ensures that the standard intrafraction preset being used for SBRT lung cases will generate an adequate number of imaging frames for 4D CT reconstruction but not greater than 1100 frames for which we would be concerned about imaging dose to the patient. Additionally, this check also helps to prevent an incorrect imaging preset from being used. This model of course is dependent on the linear accelerator model and treatment planning system being used as different machines will have different gantry rotation speeds and dose rate specifications and different treatment planning systems will optimize VMAT delivery parameters differently.

Table 2 shows the measured uncertainty in phantom target phase position determined from intrafraction 4D CBCT. Results demonstrate that the accuracy of target motion measured on this imaging modality was excellent. The average differences in target mean position, amplitude range, and standard deviation for intrafraction imaging were −0.1 ± 0.3 mm, 0.1 ± 0.5 mm and 0.0 ± 0.2 mm respectively when compared to pretreatment 4D CBCT baselines. Similar results have been reported in two other studies. The first study by Sims et. al also compared intrafraction 4D CBCT images on a lung phantom to pretreatment 4D CBCT images and found that accuracy of automatic registration of mean target position varied by ±0.9 mm (2SD).^26^ Hunt et. al evaluated intrafraction imaging on a Varian TrueBeam. Respiratory motion was simulated for a Rando phantom by moving the couch using XML‐programmed trajectories. In their study, they report a mean difference in target position of <0.4 mm and an average standard deviation of 0.6 mm.^23^ Similarly, when intrafraction images were acquired for three SBRT lung patients, measured amplitude and standard deviation results (Table 3) and target tracking shapes (Fig. 8) were also found to be in good agreement with values characterized on pretreatment 4D CBCT.

Figure 6(a) and 6(b) present the relationship between the measured uncertainty in target tracking (mean position, standard deviation, and amplitude) and treatment beam gantry span as well as treatment beam MU. The treatment beam gantry spans tested ranged from 150°–200° with more than half of the beams tested having a gantry span of close to 180°. It should be noted that if the MV gantry span were to fall outside those specified by the presets in Table 1, a new preset would need to be created in order to reconstruct kV intrafraction images for the full MV delivery. From our testing, if a new preset is not created to fully encompass the MV gantry span, kV imaging will stop at the gantry angle specified in the preset and reconstruction will begin immediately while MV treatment continues. Focusing on any single phantom setup, S20M10 for example, shows no real trend between measured uncertainty and MV gantry spans tested. This was surprising given the fact that during an intrafraction 4D CBCT acquisition, the gantry speed for gantry angles that fall outside those needed for treatment delivery is automatically set to the faster speed used for conventional 3D CBCT image acquisition (180°/min vs 67°/min typically used for *Symmetry* acquisitions). Projections acquired during these kV only gantry spans may be less uniform and may not have enough sampling at each of the 10 breathing phases and this can impact 4D CBCT image quality. In our study, the maximum kV only gantry span for the beams tested was 50° out of a total gantry span of 200° of which 58 kV projections were acquired. From Table 2, image quality for that beam was still adequate enough to determine target motion for all the phantom setups tested with a maximum absolute uncertainty of only 0.8 mm for target mean position, standard deviation and amplitude. However, intrafraction 4D CBCT images acquired for smaller MV arc lengths could be significantly impacted. This is because a large portion of the total imaging gantry span will be acquired kV‐only, using the 180°/min gantry speed.

Figure 6(b) demonstrates that larger MU treatment beams (>1500 MU) showed smaller target tracking uncertainty. These relationships make sense as large MU treatment beams with a higher MU/degree will have slower gantry rotation speeds resulting in a higher number of kV projections acquired for image reconstruction. This point is supported by the actual acquisition interval obtained for each of the 16 treatment beams tested. The acquisition interval for the largest MU beam was 0.18°/frame compared to 0.35°/frame for the smallest MU beam. This is a difference of about a factor of 2, meaning that for large MU beams almost double the number of kV projections will be acquired.

The varying number of kV projections for intrafraction imaging as well as the nonuniform gantry spacing at which projections are acquired was shown to affect the reconstructed phantom target shape (Fig. 7). While these changes in reconstructed tumor shape did not impact the accuracy of any of the target motion parameters assessed in this study (mean position, amplitude, and standard deviation), we expect that these changes would have an impact on any deformable image registration and deformable dose accumulation evaluations performed with these images. Further study on these effects is currently under our investigation.

Ideally, all MV only frames should be excluded from the phase sorting and reconstruction process. However, upon review of these XML files, it was found that those imaging frames were not being excluded and a reconstruction workaround was developed. The impact of this workaround was assessed for phantom and patient images. Errors in phase sorting for phantom images without the workaround were quite limited. The insignificance of including the MV frames in the phase sorting process for phantom images may be attributed to the construction of the lung phantom which has significantly higher contrast differences between the target volume and high‐density bolus (pseudo‐diaphragm) materials against the adjacent lung phantom material than is seen in lung patients. This allows for a reasonably accurate phase sorting process and reconstructed intrafraction 4D CBCT. However, errors in phase sorting for patient images without the workaround were significant [Figs. 10(a) and 10(b)] resulting in blurring of the diaphragm position in the reconstructed 4D CBCT phase images (Fig. 11), all of which were corrected through the implementation of the workaround. This example strongly demonstrates that modifying the XVI internal file, _frames.xml prior to sorting and reconstruction is necessary. Clinically, we have mandated that this workaround be performed for all intrafraction 4D CBCT imaging. The workaround is easy to handle for the therapists, and it would cost an additional one minute patient treatment time.

## 5 CONCLUSIONS

We have conducted a systematic study of the Elekta XVI intrafraction 4D CBCT imaging module for SBRT lung patients and validated this imaging technique with phantom and patient measurements. Through this work, we determined that a standard intrafraction imaging preset using an AcquisitionInterval parameter of 0.1°/frame can be used safely for all SBRT lung patients, greatly simplifying clinical implementation of this new imaging technique. The number of kV projections varies with MV treatment delivery parameters, however even images with low kV projection number (~550) still provided accurate target position information. An investigation into the XVI internal files revealed that some of the MV only projections acquired to remove MV scatter artifacts from images were not being excluded. A reconstruction workaround was presented and significant improvement in target tracking and image quality for patient images was demonstrated. This workaround is currently mandated for all 4D CBCT intrafraction imaging performed at our institution.

## CONFLICT OF INTEREST

The authors have no relevant conflict of interest to disclose.
